# Effects of government cash subsidies on health risk behaviors of the rural elderly: Evidence from social pension expansions in China

**DOI:** 10.18332/tid/132859

**Published:** 2021-02-02

**Authors:** Zili Zhang, Shizheng Dong, Xuanxuan Zhang

**Affiliations:** 1 School of International Trade and Economics, University of International Business and Economics, Beijing, China

**Keywords:** government cash subsidies, health risk behaviors, rural elderly, China

## Abstract

**INTRODUCTION:**

There is relatively little research on the impact of government cash subsidies on health risk behaviors of the elderly in China. We thus analyzed the effect of pension subsidies on the smoking and drinking behaviors of rural elderly using a pension scheme introduced in rural China in 2009.

**METHODS:**

Based on panel data from the Chinese Longitudinal Healthy Longevity Survey (CLHLS) in 2008 and 2011, a Difference-in-Differences (DID) method was applied to comprehensively analyze the impact of the new agricultural insurance on the health risk behaviors of the rural elderly. In order to solve possible sample selection biases, the Propensity Score Matching with Difference-in-Differences (PSM-DID) approach was used.

**RESULTS:**

We found that the implementation of the government cash subsidies clearly promoted smoking rather than drinking behavior among rural older adults. Specifically, the government cash subsidies facilitated smokers to smoke an additional 2.9 cigarettes/day, and the impact of government cash subsidies on the average cigarettes/day among smokers was more pronounced among the male elderly, lower age elderly, higher income elderly, and elderly with intact instrumental activities of daily living (IADL).

**CONCLUSIONS:**

In order to reduce the negative externalities of old-age subsidies, the government should place some restrictions on the use of cash subsidies for tobacco purchase by the elderly.

## INTRODUCTION

The relationship between cash subsidies and healthy behavior is not fully understood. On the one hand, cash subsidies can support preventive health care and healthy lifestyles^1,2^. On the other hand, cash subsidies may also support the consumption of tobacco and alcohol. It is well known that tobacco and alcohol use are major contributors to chronic diseases^3-5^. Both behaviors are not only harmful to individual health, but also increase the medical burden on society^6-8^. If government cash subsidies promote the use of alcohol and tobacco, the government needs to take certain measures for the health of the population.

Only a few studies have assessed the effects of government cash subsidies on health risk behaviors, and the conclusions are not consistent. Some studies have concluded that government cash subsidies have no or a negative impact on alcohol and tobacco consumption^9-11^. For example, Myerson et al.^11^ found that government pension subsidies significantly increased the income of the elderly but had no effect on alcohol and tobacco consumption by using a regression discontinuity design. However, some other studies argued that government cash subsidies may increase health risk behaviors^12-14^, and the effect of cash subsidies varied among different families. Case and Deaton^10^ found that households headed by females consumed relatively less alcohol and tobacco after receiving government pension benefits compared to other households. The inconsistency in the findings may be due to differences in policies across countries, or differences in the data and methods. To address this gap, we explored the effect of government cash subsidies on the smoking and drinking behavior of rural elderly in China. The expansion of China’s New Rural Social Pension Insurance (NRSPI) provided a quasi-natural experiment for our study.

In 2009, China officially started the pilot work of the NRSPI program, and expanded it to the whole country in 2012. Every rural resident aged >60 years, who is enrolled in the NRSPI, can automatically receive a minimum monthly basic pension allowance of 55 RMB (8.73 US$, exchange rate in 2011)^15^. In 2011, the NRSPI monthly subsidy amounted to 5.89 (55/9.33)^16^ times the average single-pack cigarette price and accounted for 9.46% (55/581.44)^17^ of the national monthly rural per capita disposable income. Since 2009, the new rural insurance scheme has been piloted in various regions and the number of insured persons has increased rapidly. By the end of 2011, the number of insured persons had reached about 0.33 billion, accounting for about 49.7% of the rural population^18^. The data from the 2008 and 2011 Chinese Longitudinal Healthy Longevity Survey (CLHLS) for the elderly, used in the present study, cover the period before and after the implementation of insurance policies, providing the basis for our empirical analysis. Dividing the data into treatment and control groups was a prerequisite for the use of the DID method. Respondents who did not join NRSPI during 2008–2011 were categorized into the control group. The others who did not join NRSPI in 2008, but joined NRSPI in 2011 were categorized as the treated group.

Compared with previous research, our contribution to the existing literature is in the following: 1) we examine the effect of government cash subsidies on smoking and alcohol participation among rural elderly, and we also fill a gap in knowledge of the effects of government cash subsidies on average cigarettes/day and alcohol drunk per day by rural elderly; 2) we use the PSM-DID method to minimize the problem of sample selection bias; and 3) we used the NRSPI cash subsidy program as a quasi-natural experiment that had the largest group of beneficiaries^19^. The results of the study can provide valuable information for other countries, especially those with ageing populations.

## METHODS

### Study design and setting

Data for this study were from the Chinese Longitudinal Healthy Longevity Survey (CLHLS), which was co-chaired by the Peking University Health Ageing and Development Research Center and Duke University Ageing Development Research Center. It was organized and implemented by the National Center for Disease Control and Prevention^20^. This survey was conducted in a randomly selected half of the counties and cities in 22 provinces (out of a total of 31 provinces), covering 85% of the total population in China^21^. The questionnaire included information about the elderly’s sociodemographic characteristics, insurance information, economic status, health status and family background, etc.

We obtained panel data by combining the CLHLS data from the 2008 and 2011 periods. Of the 10202 rural elders surveyed in 2008, 5206 respondents survived until 2011 and were surveyed again. In rural China, the types of pension insurance mainly include Old Rural Social Pension Insurance, commercial pension insurance, employee pension insurance and New Rural Social Pension Insurance. So, for the rural elderly who had social pension insurance and were not covered by an employee pension insurance, commercial pension insurance or Old Rural Social Pension Insurance, we considered them to be covered by the NRSPI in 2011. In order to isolate the effect of the NRSPI from other types of pension effects, we excluded the respondents with an employee pension or commercial pension insurance, as well as those who had joined the Old Rural Social Pension Insurance in 2008. To eliminate the interference of medical insurance, we further deleted participants who changed their medical insurance during 2008–2011. Ultimately, we used a sample of 8634 participants that included information on 4317 rural elderly respondents (942 and 3375 in the insured and uninsured groups, respectively) for two years. We have use large-scale survey data published in China and we are responsible for the authenticity and completeness of the data and results.

### Definitions

The variables considered in this study consist mainly of respondents’ smoking and alcohol consumption. Specifically, the variables include: current smoker (current smoking takes a value of 1, otherwise 0); cigarettes/day; current drinker (current drinking takes a value of 1, otherwise 0); and alcohol drunk per day (mainly liquor, in units of Liang, 1 Liang = 50 g).

The core explanatory variable is *NRSPI_i_* or *Treatment_i_* × *Policy_t_*, indicating whether the respondent *i* was affected by the NRSPI policy or not in the time *t*. Both *Treatment_i_* and *Policy_t_* are dummy variables. Treatment_i_ indicates whether respondent *i* participated in the NRSPI. Treatment_i_ takes the value of 1 if respondents were insured by NRSPI in 2011, otherwise 0. *Policy_t_* indicates before and after the NRSPI policy implementation, with a value of 1 for 2011 and a value of 0 for 2008.

We have controlled for other variables that affect health risk behaviors in older adults, as much as possible. Our three main categories of control variables are: sociodemographic characteristics, social support and economic conditions, and health and disease status. Sociodemographic variables include gender (1=male, 0=female), age, ethnicity (1=Han, 0=other), education years, job (1=worked in a professional or managerial position before retirement, 0=other) and married (1=married, 0=other). The social support and economic conditions variables were mainly measured by live-with-child (1=live with child, 0=other), house owner (1=house owner, 0=other) and Lnincome (logarithm of per capita household income). Health and disease status variables include IADL (the instrumental activities of daily living), self-report health, and medical expenses (the logarithm of the sum of respondents’ outpatient and inpatient costs for the past year). IADL is one of internationally accepted indicators to measure the physical health of the elderly^22^. Eight IADL activities are included: cooking, laundry, going out, visiting a home, shopping, walking, heavy lifting, squatting, and taking the bus. If an elderly person could take care of himself/herself in all these eight areas, he/she was considered to have an intact IADL (IADL=1), otherwise IADL=0. Health was based on responses to the question: ‘How do you feel about your health now?’. We grouped ‘very good’ and ‘good’ as self-rated good health (assigned a value of 1), ‘fair’, ‘bad’, and ‘very bad’ were combined with self-rated poor health (assigned a value of 0), and ‘unable to answer’ was considered as missing.

### Statistical analysis

An ordinary least squares (OLS) regression was used for the benchmark analysis. Since the endogenous problem in ordinary least squares (OLS) regression has often been pointed out^23,24^, difference-indifferences (DID) methods, and propensity score matching (PSM) methods were used in this study to address the endogeneity problem such as omitted variables and sample selection bias.

Specifically, the equations for the OLS regression analysis were as follows.

Y_it_=α_0_+α_1_NRSPI_it_+α_2_ χ_it_+ε_it_

where the dependent variable *Y_it_* represents the health behaviors of an individual *i* in year *t*. The explanatory variable *NRSPI_it_* indicates whether respondent *i* had participated in the NRSPI in year *t*. *NRSPI_it_* takes the value of 1 if respondents were insured by NRSPI in year *t*, otherwise 0; *α_1_* represents the treatment effect of NRSPI subsidies on health risk behaviors; *χ_it_* represents a set of control variables, including gender, age, ethnicity, education years, job, married, Lnincome, live-with-child, house owner, IADL, selfreport health, and medical expenses; and *ε_it_* is the error term.

A DID estimation by entity and time fixed effects regression model was then used. Entity and time fixed effects can control for the problem of omitted variables that did not vary with individuals and time. Referring to previous literature^25^, we established the following expression:

Y_it_=β_0_+β_1_Treatment_i_+β_2_Policy_t_

+β_3_ (Treatment_i_×Policy_t_)+β_4_ χ_it_+u_i_+τ_t_+ε_it_

where the dependent variable *Y_it_* now represents the health risk behaviors of an individual *i* during year *t*. The explanatory variable *Treatment_i_* indicates whether respondent *i* had participated in the NRSPI. *Policy_t_* is a dummy variable with a value of 1 in 2011 and a value of 0 in 2008; *χ_it_* is a set of control variables including age, Lnincome, live-with-child and IADL, self-report health, medical expenses; *u_i_* and *τ_t_* are the entity and time fixed effects, respectively, and *ε_it_* is the error term; *β_3_* represents the treatment effect of NRSPI subsidies on health risk behaviors and was the focus of our attention.

In addition, we used the PSM method to better reduce selectivity bias. We applied this approach in two main steps. The first step was to match propensity values for the treatment and control groups in the 2008 data, and to delete the data that were not successfully matched. The second step was to form a balanced panel of data for DID analysis with the matched 2008 data and the 2011 data using the ID of the respondent. The policy effect was the average treatment effect on the treated group (ATT). Heckman et al.^26^ demonstrated that ATT can be estimated based on the following equation:

$$ATT=E_{p(xi)|Di=1}\{E(Y_{i,post}^{p}-Y_{i,pre}^{p}|P(x_{i}),D_{i}=1)-E(Y_{i,post}^{NP}-Y_{i,pre}^{NP}|P(x_{i}),D_{i}=0)\}$$

where *P(χ_i_)=Pr(D_i_=1|χ_i_)* is the propensity score function, which is the probability that an individual *i* will participate in NRSPI given a set of observable characteristics *χ*. We chose the logit model: the explained variable is *D_i_*, and the explanatory variables are variables that affect both enrollment status *D_i_* and health risk behavior *Y_i_*, such as gender, age, education years, and health status. After estimating each individual’s propensity score, the common nearest neighbor matching method was used. We selected individuals who fell in the ‘common support’ propensity score range and matched each participant with one or more non-participants whose propensity score was sufficiently close to that of the participant. Since the ratio of treatment and control group data was 1:3.58 in this study, in order to retain more data, we used a matching ratio of 1:3. Our matching error range took a value of 0.01, which meant that if a 0.01 interval of a unit in treatment group covers one or more units in the control group, the nearest three are considered as matched units. Data that did not match were removed before we performed the DID regression analysis.

## RESULTS

Table 1 presents the descriptive statistics of the variables before propensity score matching. Column (1) reports descriptive statistics for the full sample. Columns (2) and (3) report the descriptive statistics for the treatment and control groups in 2008, and we found significant differences in many indicators between the treatment and control groups. Columns (4) and (5) report the descriptive statistics for the treatment and control groups in 2011. In terms of dependent variables, there was a decreasing trend in the number of smokers and drinkers from 2008 to 2011. Among the current smokers, the increase in the number of cigarettes/day was significantly higher in the treatment group than in the control group between 2008–2011. In terms of control variables, there was no significant difference in per capita income between the insured and non-insured groups in 2008, but in 2011, the insured group had a significantly higher income than the non-insured group. This meant that the insurance cash subsidies from the government significantly increased the income of the insured. A simple statistical description indicated that the characteristics of the treatment and control groups differed significantly before the NRSPI policy.

**Table 1:** Descriptive statistics by wave and treatment status

*Variables*	*Total*	*Wave 2008*		*Wave 2011*	
	*(1) All sample (n=8634) Mean (SD)*	*(2) Treated (n=942) Mean (SD)*	*(3) Control (n=3375) Mean (SD)*	*(4) Treated (n=942) Mean (SD)*	*(5) Control (n=3375) Mean (SD)*
**Dependent variables**					
Current smoker	0.19 (0.39)	0.22 (0.41)	0.20 (0.40)	0.20 (0.40)***	0.17 (0.38)
Cigarettes/day	11.34 (8.62)	9.91 (7.04)	10.54 (7.86)	13.05 (8.45)	12.56 (9.98)
Current drinker	0.18 (0.39)	0.24 (0.43)***	0.19 (0.39)	0.19 (0.36)***	0.16 (0.36)
Alcohol drunk per day	1.65 (2.02)	2.52 (1.97)	2.74 (2.17)	2.88 (2.26)	2.91 (2.45)
**Control variables**					
Gender	0.41 (0.49)	0.43 (0.50)*	0.40 (0.50)	0.43 (0.50)*	0.40 (0.50)
Age (years)	84.4 (11.37)	82.1 (11.18)**	83.1 (11.28)	85.1 (11.15)**	86.11 (11.27)
Education years	1.52 (2.60)	1.67 (2.65)**	1.48 (2.58)	1.67 (2.65)**	1.48 (2.58)
Job	0.04 (0.18)	0.04 (0.19)	0.04 (0.18)	0.04 (0.19)	0.04 (0.18)
Ethnicity	0.93 (0.26)	0.97 (0.18)***	0.91 (0.28)	0.97 (0.18)***	0.91 (0.28)
Married	0.36 (0.48)	0.40 (0.49)	0.37 (0.48)	0.37 (0.48)***	0.32 (0.47)
Lnincome	7.68 (1.83)	7.68 (1.39)	7.60 (1.56)	7.98 (1.74)***	7.67 (2.17)
Houseowner	0.43 (0.49)	0.48 (0.49)	0.47 (0.49)	0.40 (0.49)	0.38 (0.49)
Live with child	0.51 (0.50)	0.49 (0.50)	0.49 (0.50)	0.51 (0.50)*	0.54 (0.50)
Self-report health	0.44 (0.50)	0.53 (0.50)***	0.46 (0.50)	0.41 (0.49)	0.39 (0.49)
IADL	0.37 (0.48)	0.44 (0.50)*	0.41 (0.49)	0.37 (0.48)***	0.31 (0.46)
Medical expenses	4.8 (3.08)	4.79 (2.66)	4.74 (2.72)	5.22 (3.17) ***	4.78 (3.46)

*** p<0.01,

** p<0.05 and

* p<0.1; from t-tests for the control and treatment group variables in each wave. Lnincome: logarithm of per capita household income. SD: standard deviation.

Part A of Table 2 reports the mean values, bias and t-test of the variables in the treatment and control groups before and after PSM. Compared with the prematch (Unmatched) data, the standardized deviations of the characteristic variables between both insured and non-insured groups were substantially reduced in the post-match (Matched) data. The t-test results for each indicator after matching were not significant, indicating that the original hypothesis that the covariates were not significantly different between the insured and non-insured groups was not rejected. Part B of Table 2 reports the pre-match and post-match mean bias of all variables (7.3 vs 1.1). Overall, the differences between the treatment and control groups were no longer significant after matching.

**Table 2:** Balancing tests from neighbor matching

*A. Test of the balancing property for each observed covariate*							
*Variables*	*Unmatched*	*Mean*		*Bias*		*t-test*	
	Matched*	*Treated*	*Control*	*Bias %*	*Reduct bias %*	*t*	*p*
Gender	U	0.435	0.404	6.4		1.73	0.083
	M	0.437	0.425	2.9	54.1	0.63	0.527
Age (years)	U	82.063	83.084	-9.1		-2.46	0.014
	M	82.063	82.19	-1.1	87.5	-0.25	0.805
Ethnicity	U	0.967	0.911	23.5		5.71	0.000
	M	0.967	0.967	0.1	99.4	0.04	0.966
Education years	U	1.673	1.484	7.2		1.97	0.049
	M	1.673	1.65	1.0	86.6	0.20	0.838
Job	U	0.037	0.034	0.9		0.26	0.799
	M	0.037	0.036	-1.2	-23.2	-0.24	0.808
Married	U	0.406	0.375	6.6		1.79	0.073
	M	0.406	0.405	0.4	93.7	0.09	0.928
Lnincome	U	7.685	7.601	6.2		1.60	0.110
	M	7.690	7.676	0.9	84.6	0.20	0.838
Houseowner	U	0.488	0.469	3.9		1.05	0.295
	M	0.488	0.485	0.6	83.5	0.14	0.890
Live with child	U	0.494	0.491	0.5		0.14	0.885
	M	0.494	0.498	-1.1	-99.0	-0.23	0.818
Self-report health	U	0.539	0.462	15.5		4.21	0.000
	M	0.539	0.548	-1.8	88.6	-0.39	0.700
IADL	U	0.445	0.412	6.6		1.80	0.073
	M	0.445	0.441	0.8	88.6	0.16	0.871
Medical expenses	U	4.785	4.746	1.5		0.39	0.699
	M	4.785	4.745	1.5	-3.9	0.32	0.746
*B. Test of the overall balance*							
*Sample*	*LR χ^2^*	*p>χ^2^*		*Mean bias*		*Median bias*	

Unmatched	67.55	0.000		7.3		6.5	
Matched	0.95	1.000		1.1		1.0	

* Unmatched (U) are the data before the smoking and non-smoking groups were matched. Matched (M) are the data after the match. Lnincome: logarithm of per capita household income.

Table 3 presents the regression results of the impact of NRSPI cash subsidies on health risk behaviors of the rural elderly. The OLS, DID, and PSM-DID methods produce generally consistent results, indicating that the conclusions are robust. In terms of tobacco use, regression results from different models showed that cash subsidies did not have a significant effect on smoking prevalence, but promoted a significant increase in average cigarettes/ day among current smokers. In terms of alcohol use, we found no significant effect of NRSPI cash subsidies on drinking behavior among the elderly.

**Table 3:** The impact of NRSPI cash subsidies on health risk behaviors

Characteristics	OLS	FE	PSM-DID
Current smoker	0.0154 (0.0134)	0.0111 (0.0134)	0.0111 (0.0146)
N	7939	7939	5072
R^2^	0.1866	0.0037	0.0053
Cigarettes/day	2.266 (0.663)***	2.307 (0.964)**	2.926 (1.056)***
N	1714	1714	1168
R^2^	0.0782	0.0296	0.0491
Current drinker	0.0110 (0.0139)	-0.0170 (0.0169)	-0.0145 (0.0182)
N	7939	7939	5072
R^2^	0.1103	0.0083	0.0103
Alcohol drunk per day	0.276 (0.176)	-0.0931 (0.283)	0.125(0.334)
N	1464	1464	984
R^2^	0.0938	0.0276	0.0310

Robust standard errors in parentheses. *p<0.1,

** p<0.05,

*** p<0.01. All other control variables are controlled for.

Considering the potential heterogeneous effects of the government cash subsidies on different cohorts, we disaggregated the sample by gender, age, income, and IADL status in the baseline period of 2008. The heterogeneity analysis was mainly based on the PSMDID method due to its advantages over other methods, and the regression results are shown in Table 4. The results of the heterogeneity analysis showed that government cash subsidies had no significant effect on smoking initiation among rural elderly in each group, but had a significant effect on the average cigarettes/ day among smokers. Specifically, the impact was more pronounced among male elderly, lower age elderly, higher income elderly, and elderly with intact IADL. In addition to the above findings, the results of the heterogeneity analysis in Table 4 show that none of the coefficients on the interaction terms is significant when the explanatory variable is drinking behavior, indicating that the NRSPI cash subsidies had no effect on the drinking behavior of the rural elderly.

**Table 4:** Results of the heterogeneity analysis

	*Gender*		*Age (years)*		*Lnincome*		*IADL*	
	*(1)*	*(2)*	*(3)*	*(4)*	*(5)*	*(6)*	*(7)*	*(8)*
	*Male*	*Female*	*60–79*	*≥80*	*High income*	*Low income*	*IADL=1*	*IADL=0*
Current smoker	0.0420	-0.0099	0.0250	0.0082	0.0301	-0.0075	0.0291	-0.0043
	(0.0293)	(0.0131)	(0.0261)	(0.0185)	(0.0213)	(0.0201)	(0.0224)	(0.0195)
N	2146	2926	1914	3158	2554	2518	2223	2849
R^2^	0.0130	0.0069	0.0094	0.0072	0.0033	0.0176	0.0084	0.0065
Cigarettes/day	2.166*	7.128***	3.256*	2.419*	3.614**	2.406*	3.176**	1.716
	(1.165)	(2.691)	(1.706)	(1.421)	(1.540)	(1.453)	(1.339)	(1.748)
N	965	203	569	599	581	587	700	468
R^2^	0.0450	0.2443	0.0569	0.0860	0.0746	0.0967	0.0521	0.1083
Current drinker	0.0122	-0.036**	-0.0183	-0.0137	-0.0033	-0.0246	-0.0309	-0.0043
	(0.0345)	(0.0183)	(0.0307)	(0.0237)	(0.0265)	(0.0249)	(0.0287)	(0.0233)
N	2146	2926	1914	3158	2554	2518	2223	2849
R^2^	0.0343	0.0071	0.0252	0.0107	0.0142	0.0162	0.0222	0.0071
Alcohol drunk per day	0.153	0.112	0.440	-0.116	0.484	0.107	0.285	0.130
	(0.414)	(0.243)	(0.515)	(0.435)	(0.516)	(0.411)	(0.463)	(0.340)
N	727	257	451	533	482	502	549	435
R^2^	0.0345	0.1110	0.0781	0.0267	0.1211	0.0805	0.0338	0.1133

Lnincome: logarithm of per capita household income. Robust standard errors in parentheses.

* p<0.1, *p<0.05,

*** p<0.01. All other control variables were controlled for. ‘High income’ denotes the elderly whose per capita household income was higher than average income in 2008, and ‘Low income’ denotes the elderly whose per capita household income was lower than average income in 2008.

## DISCUSSION

This study is the first in China to use panel data to examine the impact of pension subsidies on the average cigarettes/ day and daily alcohol intake among rural elderly. Our findings contribute to the literature on the impact of cash subsidies on health-related behaviors of the elderly.

The overall sample estimates from the PSM-DID method showed that the NRSPI cash subsidy resulted in an average of 2.9 additional cigarettes/day by insured smokers. The possible reason is that rural elderly can have access to a stable income stream after they joined in the NRSPI, which can ease the budgetary constraints of the rural elderly and increase their consumption levels^27,28^. The increased cigarette affordability of the insured elderly contributed to their increased cigarette consumption. At the end of 2011, the number of elderly people in rural areas receiving NRSPI cash subsidies was 89.22 million^29^. Estimating a 22% smoking participation rate of the insured elderly in the research sample, an increase of 2.926 cigarettes/day among smokers due to NRSPI cash subsidies would result in an additional consumption of over 57 million (89.22 × 22% × 2.926 = 57.43 million) cigarettes/day in China. The large additional cigarette consumption would result in a serious direct and indirect disease burden.

Unlike smoking behavior, we found no significant effect of NRSPI cash subsidies on drinking behavior among the elderly. There are two possible explanations. On the one hand, the dangers of alcohol consumption for older people are more rapid and immediate than those of smoking, for example, alcohol consumption not only increases the risk of falls in older people, it also prevents many drugs from working or has side effects^30^. Older people are generally more likely to become ill and therefore need to take medication^31^. This partly inhibited the incentive to consume alcohol that came with increased income of the elderly. On the other hand, drinking alcohol was more likely to occur among a group of people than smoking^32^. In fact, the small amount of cash subsidies from the NRSPI was not enough to support the consumption of alcohol by groups. Thus, the above two factors can be used to partially explain why the NRSPI cash subsidy had a more pronounced effect on cigarette consumption than on alcohol consumption.

The effect of government cash subsidies on health risk behavior was heterogeneous across different categories of people. First, the NRSPI cash subsidies both significantly increased average cigarettes/day among rural male and female elderly, but the increase was more pronounced for the female elderly. In rural China, the income of a family is mainly earned by men, who dominate the household, which may lead to more limitations for women compared to men in terms of expenditure. Under this assumption, cash subsidies may ease the consumption constraint to a greater extent for female elderly than for male elderly, and stimulate an increase in the average cigarettes/day among rural females. Second, compared to the low-income group, the elderly in the high-income group showed a more pronounced increase in smoking behavior affected by the pension cash benefit. The possible reason is that subsidized income has different utility for people with different incomes, so their consumption behavior using the subsidy may also be different^33^. Third, when analyzed by different IADL status, insured smokers with better IADL status had an average increase of 3.2 cigarettes/day. In contrast, the increase in smoking among insured smokers with poorer health status was not significant. This heterogeneity may be due to the fact that older adults in poorer health visited the doctor frequently, and visiting clinics or hospitals can attenuate their health risk behaviors by acquiring knowledge of effective disease prevention and enhancing their awareness of the hazards of unhealthy behaviors^34^. Fourth, the results by age showed that lower age elderly smokers who received the subsidy increased the cigarettes/day more than higher age elderly smokers. This finding is actually consistent with the findings by IADL, as older seniors generally had lower IADL than younger seniors.

### Limitations

This study has some limitations. As with other survey data, this research was limited to variables that were available in our data. We have no information about hospitalization of the rural elderly, so it would be hard to study the clinical impact on health of cigarette smoking. Moreover, regrettably, we could only use the Chinese survey data of 2008 and 2011 to find causal relations between cash subsidies and health behaviors of the rural elderly. So, care is needed when extrapolating our findings from China to other countries. Our conclusions are limited based on self-reported results from the CLHLS data, and our findings apply only to rural elderly aged >60 years. In the future, if data become available, we will examine the impact of government cash subsidies on risk behavior in other populations.

## CONCLUSIONS

The purpose of the government’s NRSPI policy was to protect the lives of rural residents. Many researchers have confirmed that government pension subsidies improved the quality of life of rural people and reduced their incidence of poverty^15,19,29^. However, the government also needs to be aware of the negative externalities caused by government pension subsidies. Using CLHLS data and a natural experiment with NRSPI expansion, the results of the DID and PSMDID methods showed that government cash subsidies promoted an increase in the average cigarettes/day among the insured rural elderly smokers. In the context of healthy ageing, governments around the world should place some limits on the use of government cash subsidies for the purchase of tobacco products.

## CONFLICTS OF INTEREST

The authors have completed and submitted the ICMJE Form for Disclosure of Potential Conflicts of Interest and none was reported.

## FUNDING

This work was supported by the National Natural Science Funds of China (71373045).

## AUTHORS’ CONTRIBUTIONS

Conceptualization: ZZ, XZ. Methodology: ZZ, SD. Software: ZZ, XZ and SD. Validation: ZZ, SD. Formal analysis: ZZ, XZ and SD. Writing of original draft: ZZ, XZ. Manuscript review and editing: XZ, SD. Supervision: XZ, SD. Project administration: ZZ, XZ and SD. All authors read and approved the submitted manuscript.

## PROVENANCE AND PEER REVIEW

Not commissioned; externally peer reviewed.
